# Supplementary figures and images for: The role of inoculum dispersal and plant species identity in the assembly of leaf endophytic fungal communities
Source: PLoS One. 2019 Jul 16;14(7):e0219832. doi: 10.1371/journal.pone.0219832 (PMC6640817; doi:10.1371/journal.pone.0219832)

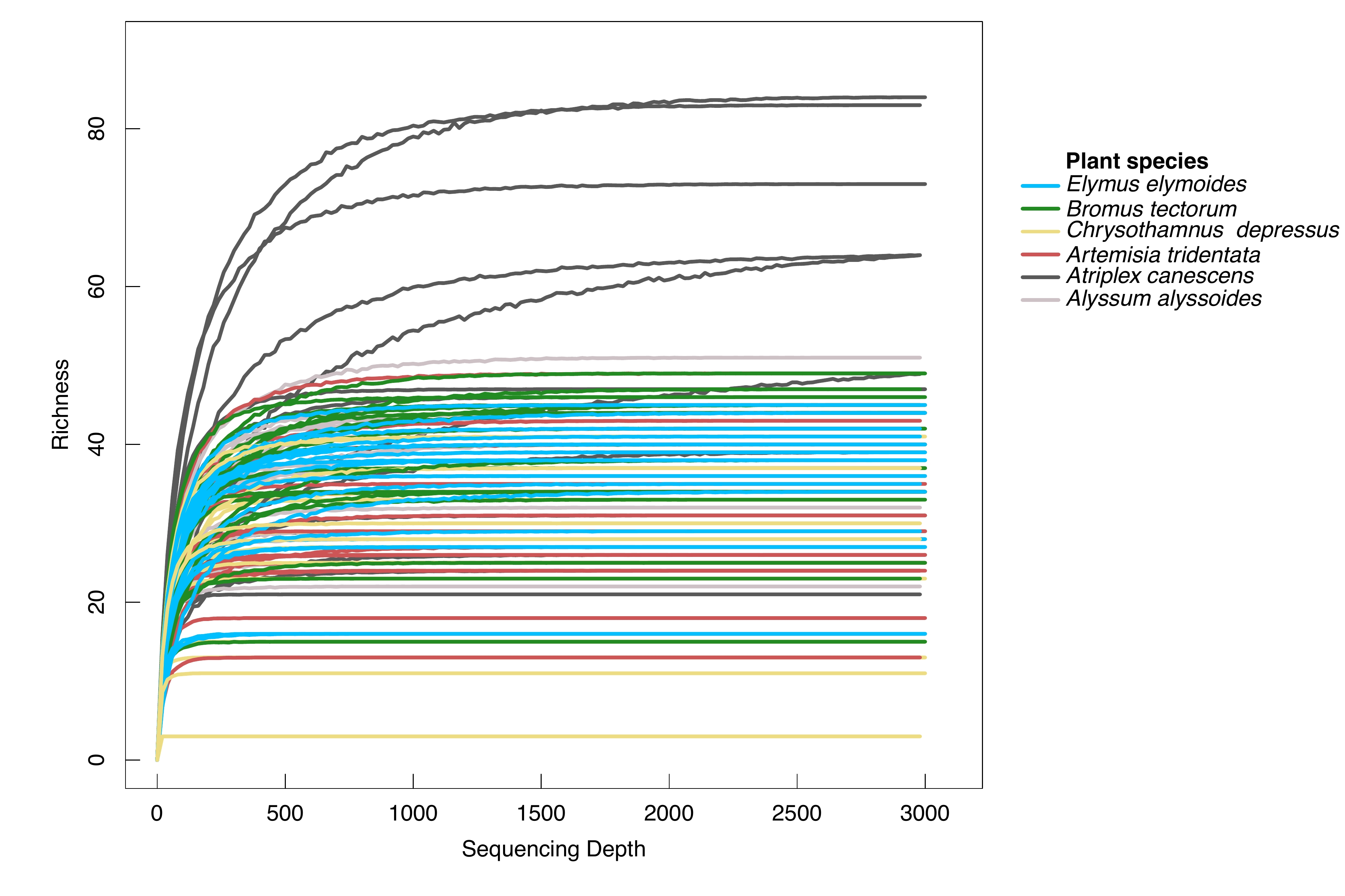

Supplement: S1 Fig — All samples have been rarefied to an equal depth of 3,000 reads. Each line represents an individual sample. Curves were generated using the rarefy function in the Vegan package [51]. (TIF) [file pone.0219832.s001.tif]
