# Supplementary material for: The role of inoculum dispersal and plant species identity in the assembly of leaf endophytic fungal communities
Source: PLoS One. 2019 Jul 16;14(7):e0219832. doi: 10.1371/journal.pone.0219832 (PMC6640817; doi:10.1371/journal.pone.0219832)
Supplement: Attachment — Submitted filename: Responses to reviews.docx [file pone.0219832.s002.docx]

1. When submitting your revision, we need you to address these additional requirements.

Please ensure that your manuscript meets PLOS ONE's style requirements, including those for file naming. The PLOS ONE style templates can be found at

<http://www.journals.plos.org/plosone/s/file?id=wjVg/PLOSOne_formatting_sample_main_body.pdf> and <http://www.journals.plos.org/plosone/s/file?id=ba62/PLOSOne_formatting_sample_title_authors_affiliations.pdf>

2.

We note that you have indicated that data from this study are available upon request. PLOS only allows data to be available upon request if there are legal or ethical restrictions on sharing data publicly. For more information on unacceptable data access restrictions, please see <http://journals.plos.org/plosone/s/data-availability#loc-unacceptable-data-access-restrictions>.

Our sequence data were submitted to the sequence read archive at NCBI. We now include the accession number.

In your revised cover letter, please address the following prompts:

a) If there are ethical or legal restrictions on sharing a de-identified data set, please explain them in detail (e.g., data contain potentially sensitive information, data are owned by a third-party organization, etc.) and who has imposed them (e.g., an ethics committee). Please also provide contact information for a data access committee, ethics committee, or other institutional body to which data requests may be sent.

b) If there are no restrictions, please upload the minimal anonymized data set necessary to replicate your study findings as either Supporting Information files or to a stable, public repository and provide us with the relevant URLs, DOIs, or accession numbers. For a list of acceptable repositories, please see <http://journals.plos.org/plosone/s/data-availability#loc-recommended-repositories>.

Our sequence data were submitted to the sequence read archive at NCBI. We now include the accession number.

We will update your Data Availability statement on your behalf to reflect the information you provide.

[Note: HTML markup is below. Please do not edit.]

Reviewers' comments:

Reviewer's Responses to Questions

**Comments to the Author**

1. Is the manuscript technically sound, and do the data support the conclusions?

The manuscript must describe a technically sound piece of scientific research with data that supports the conclusions. Experiments must have been conducted rigorously, with appropriate controls, replication, and sample sizes. The conclusions must be drawn appropriately based on the data presented. 

Reviewer #1: Yes

Reviewer #2: Yes

2. Has the statistical analysis been performed appropriately and rigorously? 

Reviewer #1: Yes

Reviewer #2: Yes

3. Have the authors made all data underlying the findings in their manuscript fully available?

The [PLOS Data policy](http://www.plosone.org/static/policies.action#sharing) requires authors to make all data underlying the findings described in their manuscript fully available without restriction, with rare exception (please refer to the Data Availability Statement in the manuscript PDF file). The data should be provided as part of the manuscript or its supporting information, or deposited to a public repository. For example, in addition to summary statistics, the data points behind means, medians and variance measures should be available. If there are restrictions on publicly sharing data—e.g. participant privacy or use of data from a third party—those must be specified.

Reviewer #1: Yes

Reviewer #2: Yes

4. Is the manuscript presented in an intelligible fashion and written in standard English?

PLOS ONE does not copyedit accepted manuscripts, so the language in submitted articles must be clear, correct, and unambiguous. Any typographical or grammatical errors should be corrected at revision, so please note any specific errors here.

Reviewer #1: Yes

Reviewer #2: Yes

5. Review Comments to the Author

Please use the space provided to explain your answers to the questions above. You may also include additional comments for the author, including concerns about dual publication, research ethics, or publication ethics. (Please upload your review as an attachment if it exceeds 20,000 characters)

Reviewer #1: The authors explored the important effects of inoculum dispersal and plant species identity on community assembly of endophytic fungi. The study design is appropriate, as are the methods used, the paper is well-written and therefore I think it deserves publication in PLoS One.
Only three minor comments:
1. The letter “P” (P value) should be italic in Table 1, Table 4, Line 148, Line 164, Line 224, Line 232, Line 234.

This has been corrected.

2. “sp” should not be written in italic (Line 204-208, Line 218, Line259-274, Table 3)

This has been corrected.

3. In discussion, it would be appreciated if authors can cite of the major drawbacks encountered in this study and cite some of the future works.

Some limitations of the study are now included in the fifth paragraph of the discussion. More than half of the discussion is devoted to future work in the form of hypotheses that we feel merit testing, and the reasons why we feel they should be tested.

Reviewer #2: This manuscript investigated the effects of both plant identity and fungal inoculum dispersal limitation on endophytic fungal community structure in leaves of six plant species in the eastern Great Basin of the USA, which are interesting. I have some minor comments and suggestions before publication:
Materials and methods:
Ln 95-102: You should give more information about the plots, for example, how did you establish the plots? and the distance among plots.

These are now given in more detail in the second paragraph of the materials and methods.

In addition, it is unclear how many samples were actually sampled and sequenced. 18 replicated plots, and for each plot, a single sample for each of the six plant species, so 108 samples were collected and sampled? You should state clearly here.

That is correct. This is now specified in the second paragraph of the materials and methods.

Ln 106-107: How did you test the effectiveness of surface sterilization? Did you collect the sterile water used in the rinse and do the PCR or just culture on medium?

We tested the effectiveness of the method by determining the extent to which it and other methods were capable of reducing total fungal species richness. The sodium hypochlorite method that we used was significantly more effective than ethanol, which appears to be a popular method used by others in this field. Thank you for the suggestion about testing the sterile water rinse. However, the sterile water rinse would not have been an acceptable way to test the efficacy of the method because fungi or fungal DNA could still be adhering to the leaf surface and not appear in the rinse.

Ln 116: 5.8S Fun and ITS4 Fun

Corrected.

Ln 128-131: Why did you rarefy the samples prior to all sequence analyses?

As we specified in the text, “This made samples comparable despite the potential for different original sequencing depths.” Rarefaction is a common practice in this kind of study to ensure comparability when calculating such things as diversity, evenness, etc. because it gives each sample the same sampling depth.

I saw the Figure S1, the sequence depth is 3000. So this step is the same with or different from the rarefaction you mentioned in Ln 141-142?

Yes, this is the same and it is now more clearly specified.

Ln136-Ln139: You amplified the ITS2 region using the 5.8S Fun and ITS4 Fun. Generally, some studies will extracted ITS before the OTU cluster, Why did not you do that?

Unfortunately, we do not understand this comment

In addition, did you check the chimera? What was the proportion of chimeric sequences?

Yes. Using the UCHIME function, 0.8% of reads were identified as chimeras, and these were removed. We now include this information in the 6^th^ paragraph of the materials and methods.

Ln 140: What was the proportion of your ITS sequences belonged to the host in each sample?

6% of the total reads belonged to the host. This information is now included in the 6^th^ paragraph of the materials and methods.

Have authors deposited sequences to a public repository? You should state in the manuscript.

Yes. FASTQ files are deposited in the Sequence Read Archive (SRA) at NCBI (accession number PRJNA518913). This information is now included in the 6^th^ paragraph of the materials and methods.

Results:
Ln 174: How many raw data were obtained for each sample? And how about the reads for each sample before rarefaction?

There was an average of 12,816 per sample before filtering. After filtering and prior to rarefaction there was an average of 7167 per sample. This information is now given in the first paragraph of the Results section.

L173-176: Maybe it is better to add some description about the fungal taxonomic composition in the first paragraph. You should provide a supporting information about your fungal identification.

This description is now given in the form of a new figure (Fig 3).

Ln 215: Please cite the "Table 3" behind "among plant families"

This has been corrected.

Table 3: I think this table does not show the information visually. Maybe you can colored the cell of the table according to the frequencies of occurrence, like a heatmap.

It may be possible to turn this table into a figure as the reviewer suggests. However, we felt that more information could be conveyed by presenting the actual frequencies rather than colors coded to frequency. Our goal was to simultaneously show the frequencies, and color-code the columns so plant families were clear.

Discussion:
Ln 317-318: Are there any other publications describing the 18 fungal taxa colonizing leaves of other plant species? And are there publications showing these fungal taxa contribute to the host health and development? Maybe you can add some discussion about this.

Of the 18 fungal taxa that were found in all six species, only 7 were identifiable to species. Even in these 7 cases, it is not clear whether knowing if they colonize other plant species and influence their health in any way is useful because the effects of endophytic fungal colonization is context dependent (dependent on host species and environmental conditions) and on the specific isolate of the fungal species. Moreover, the main point of our findings is not to suggest that any of these 18 fungal species could be used to promote revegetation of the great basin plant species that we studied. The point of our study was to determine the extent to which plant identity and dispersal limitation generally influence endophytic fungal community structure. That knowledge will influence how we use inoculant fungi in the future, not necessarily which fungi we choose for that purpose.
